# Autogenous Dentin Particulate Graft for Alveolar Ridge Augmentation with and without Use of Collagen Membrane: Preliminary Histological Analysis on Humans

**DOI:** 10.3390/ma15124319

**Published:** 2022-06-18

**Authors:** Elio Minetti, Francesco Gianfreda, Andrea Palermo, Patrizio Bollero

**Affiliations:** 1 Department of Biomedical, Surgical, and Dental Science, University of Milan, 20122 Milan, Italy; elio.minetti@unimi.it; 2 Department of Industrial Engineering, University of Rome “Tor Vergata”, 00133 Rome, Italy; 3 College of Medicine and Dentistry, Birmingham B4 6BN, UK; andrea.palermo2004@libero.it; 4 Department of System Medicine, University of Rome “Tor Vergata”, 00133 Rome, Italy; patrizio.bollero@ptvonline.it

**Keywords:** autogenous dentin graft, alveolar ridge augmentation, membrane, bone graft histology, tooth transformer

## Abstract

(1) Background: The phenomenon of ankylosis of the dental elements has led clinicians to think that properly treated dentin and cement may be a potential graft for alveolar ridge augmentation. Currently, there are no studies in the literature able to histomorphometrically compare the healing patterns of an autogenous dentin particulate graft with the association, or not, of resorbable membranes. The aim of this pilot study is to histologically compare bone after an alveolar ridge augmentation using an autogenous dentin particulate graft with and without a resorbable collagen membrane. (2) Methods: this clinical trial enrolled six patients with defects requiring bone augmentation. Two procedures were performed in all six adult human patients in order to perform a study–control study: in Group 1, a ridge augmentation procedure with an autogenous dentin particulate graft and a resorbable collagen membrane was performed, and, in Group 2, an alveolar ridge preservation without a membrane was performed at the same time (T0). At 4 months, a biopsy of the bone tissues was performed using a 4 mm trephine bur in order to perform a histomorphometric analysis. (3) Results: The histomorphometric analysis demonstrated that Group 1 presented 45% of bone volume, 38% of vital bone, and 7% of residual graft. On the contrary, membrane-free regenerative procedures demonstrated 37% of bone volume, 9% of vital bone, and 27% of non-resorbed graft. In all cases, the regenerated bone allowed the insertion of implants with a standard platform, and no early failures were recorded. (4) Conclusions: Autogenous dentin particulate grafts seem to work best when paired with a membrane.

## 1. Introduction

The ability to place dental implants in sites that have undergone tooth extraction is closely related to the amount of bone after the resorption of the bundle bone [1]. Bone remodeling is a complex process that involves all the bone tissues in the organism and, obviously, could cause the resorption of alveolar bone due to the lack of trophic stimuli from the periodontal ligament [2]. The first year after the extraction, the soft and hard tissues undergo numerous dimensional variations [3]. After the extraction, the residual bone dimension is important to allow for the implant insertion [4,5,6].

Many studies provided histological evidence of bone regeneration in extraction sockets following applications of resorbable membranes in combination or in association with bone grafts of allogeneic and xenogeneic origin [7,8]. Several surgical procedures were described and validated by the scientific literature aimed at increasing the bone volume using different graft materials, classified as autograft (bone from the same patient), allograft (bone from another human), xenograft (bone from other species), and alloplast (synthetic material) [9]. Vasilic compared the clinical effectiveness of bovine porous bone mineral combined, or not combined, with an autologous fibrinogen/fibronectin system in preserving alveolar ridges following tooth extraction. The results demonstrate significantly less horizontal resorption (1.06 ± 0.28 mm vs. 2.60 ± 0.25 mm) between the two groups [9]. Barone and al. compared grafted vs. non-grafted post-extractive sockets and showed that the grafted sites allowed for longer and wider placement [10].

Tooth extraction is certainly the most performed surgical procedure in dentistry, and the teeth extracted were considered waste elements. Recent clinical studies have proposed the reimplantation of the same dental elements, the partial retention of the root to allow for the preservation of the bundle bone, and the use of particulate dentin as a graft material [11,12,13,14,15,16,17,18,19,20]. Studies in humans have demonstrated the clinical efficacy and safety of the partially demineralized autogenous dentine matrix prepared chairside as a bone graft [19,20,21,22,23]. Some authors theorized that the demineralization process of dentin allows for better bone augmentation, probably due to the fact that the exposure of these molecules can increase bone apposition [24].

A recent series of cases has shown that it is possible to perform bone regeneration using autologous tooth grafting, leading to a filling of the bone defects without complications [25].

Regeneration procedures require the use of a membrane with the aim of providing the bone tissue cells with the necessary space for bone regeneration away from the surrounding connective tissue.

Currently, there are no studies in the literature able to histomorphometrically compare the healing patterns of an autogenous dentin particulate graft with the association, or not, of resorbable membranes.

The aim of the pilot study is to establish if there are histomorphometrical and clinical differences between the use of an autogenous dentin particulate graft with and without collagen membrane after alveolar ridge augmentation procedures.

## 2. Materials and Methods

This clinical trial was conducted in accordance with Good Clinical Practice guidelines (GCP). Specifically, the World Medical Association Declaration of Helsinki, Ethical Principles for Medical Research Involving Human Subjects, as revised in Fortaleza, were followed. Patients were asked to sign an informed consent.

The Ethical Committee of the University of Chieti accepted the design of the research study with the protocol N 1869, 12 December 2018.

Operators with experience (E.M., A.P.) performed the surgical procedures.

### 2.1. Study Design

The patients selected were who required ridge augmentation on an edentulous site and on a post-extraction socket. Where ridge augmentation was required, an autogenous dentin graft was inserted with the addition of a membrane (Group 1, study group), while an autogenous dentin graft was inserted exclusively in sites with post-extraction sockets (Group 2, control group).

### 2.2. Inclusion Criteria

The study considered patients with good health status (ASA-1, ASA-2) over the age of 18. All patients required tooth extractions for periodontal reasons, trauma, or caries. The need for an alveolar ridge augmentation to maintain or increase bone volume for implant rehabilitation was considered in the inclusion criteria.

### 2.3. Exclusion Criteria

Pregnant subjects, patients with a history of allergies, tobacco use (within the last six months), diabetes, cancer, human immunodeficiency virus (HIV), metabolic and bone tissue disorders, treatment with immunosuppressive agents or use of systemic corticosteroids or antiresorptive drugs such as intramuscular/intravenous bisphosphonates, and patients on radiation therapy and chemotherapy were excluded.

### 2.4. Preoperative Work-Up

Clinical and radiographic analysis with cone-beam computed tomography (CBCT, Planmeca ProMax 3DS Helsinki, Finland), periapical X-rays, or panoramic X-rays was performed. An oral hygiene session was performed two weeks before the surgical treatments. Each patient was prescribed a 0.2% chlorhexidine-based mouthwash, twice a day, for two weeks.

### 2.5. Surgical Procedures and Follow-Up

Each patient was prescribed antibiotic prophylaxis with 2 g amoxicillin/clavulanic acid in a solution 2 h before the extractions.

All extracted teeth were decontaminated and cleaned with a diamond bur under abundant irrigation. Subsequently, each dental element was divided into samples of about 5 mm in size.

The samples obtained were inserted into the Tooth Transformer (TT) grinder device (TT Tooth Transformer srl. Milan, Italy) to carry out a demineralization treatment for 25 min.

The entire extracted tooth was cleaned with a diamond bur (ref. 6855 Dentsply Maillefer, Ballaigues, Switzerland) under abundant irrigation with physiological water. All filling materials (gutta-percha, composite, luting cements, etc.) were removed with the utmost care and under magnification. Subsequently, the tooth was cut into fragments (5 × 5 mm) and inserted into the milling device (Tooth Transformer, Milan, Italy). According to the manufacturer, a disposable box containing disposable liquid solutions is inserted into the device to ensure the demineralization of the graft with 0.1 M hydrochloric acid, 10% hydrogen peroxide, and demineralized water as a wash.

The decontamination of the granules takes place through UVA rays and ultrasounds, with temperature variations always lower than 43 °C to avoid damage to proteins.

After 25 min, particle graft biomaterials were obtained. The particles were partially demineralized by the TT device, and the average particle size varied between 406 and 815 μm with peaks up to 1110 μm [23].

All bone defects were filled with the product obtained from the TT grinder. In Group 1, the dentin graft was associated with a resorbable membrane (Group 1, graft covered with Membrane Osseoguard, Zimmer Biomet, Freiburg im Breisgau, Germany) and, in Group 2, the membrane was not placed.

**Figure 1:**
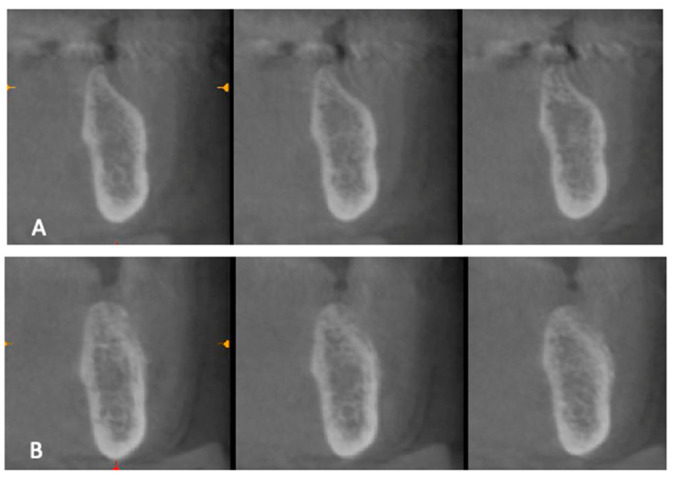
Radiographic investigation before surgery (**A**) and after the healing time (**B**) before implants.

A radiographic investigation (Figure 1) carried out after 4 months determined whether it was possible to place the implants (Visio One^®^, CEA Medical Sa, Geneva, Switzerland).

After local anesthesia, a full-thickness mucoperiosteal flap was detached in the augmented areas and an implant placement was performed. During the preparation of the implant site, a biopsy of the bone tissues was performed using a 4 mm trephine bur. The surgical procedure then required implant placement and flap closure.

A second surgery was performed when the implants healed and a screw-retained metal–ceramic prosthesis was delivered.

### 2.6. Histological Technique

The samples were decalcified, paraffin embedded, and cut. The samples were fixed in 10% neutral buffered formalin (37% formaldehyde solution 10 mL, NaCl 0.8 g, monobasic potassium phosphate 0.4 g, dibasic potassium phosphate 0.65 g, and distilled water 90 mL) for 7 days. Descaling was performed with disodium EDTA pH 7 until total descaling; the endpoint was physically determined. The samples were then dehydrated in ethanol at an increasing concentration from 70% to 100%, clarified with xylene, and embedded in paraffin; all chemical uses were made with Carlo Erba reagents. The paraffin slides were obtained with a Lecia RM2245 rotary microtome and placed on superfrost microscope slides and mounted with Biomout HM bio-optica.

The histological images obtained from the transmitted light microscope (Olympus) was digitized through a digital camera and analyzed by means of an image analysis software IAS 2000 (QEA). With the histomorphometric analysis, we distinguished:

- Bone volume % (BV%), which represented the percentage of mineralized tissue with the exclusion of medullary tissues.
- Tooth Transformer graft % (TT%), which represented the percentage of the volume occupied by the remaining graft, namely dentin.
- Vital bone % (VB%), which represented the percentage of vital bone, excluding medullary tissues.

The amount of BV% was the sum of TT% and VB%. Each section was measured using ImageJ program (version 1.8.0_72, National Institutes of Health, Bethesda, MD, USA).

## 3. Results

The six patients were two males and four women. The bone defects to be treated were all in the mandible, with the exception of one patient who required treatment in the maxilla. The average age was 55.16 ± 14.6 (from 35 to 78). The Group 1 sites were one or two wall defects; the Group 2 sites were four walls defects because they were only post extractive. All the biopsies were made after 4 months of healing.

**Figure 2:**
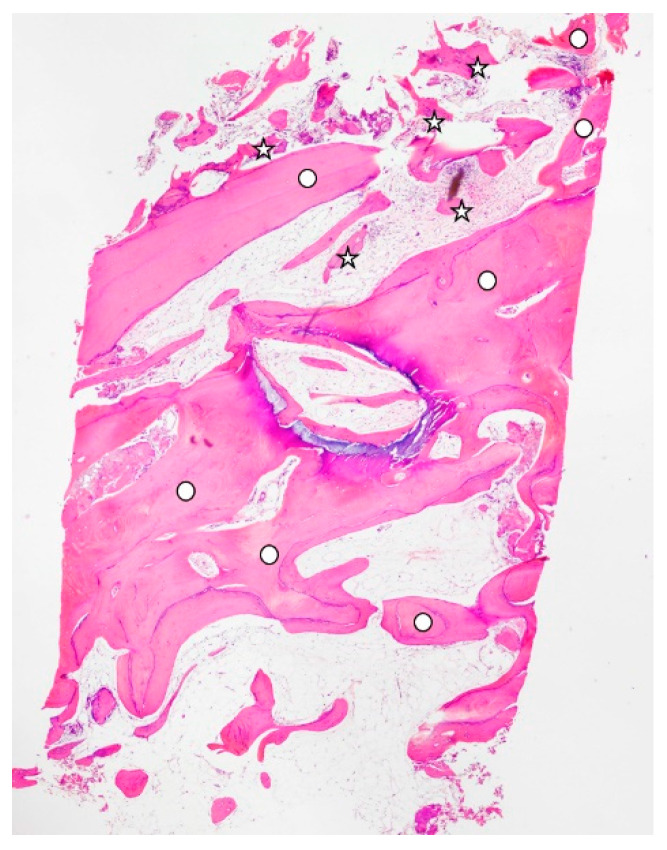
Histology extracted from a site with membrane, with a circle for the bone and a star for the tooth graft. New bone was 41.67%, residual graft was 5.65%, and total calcificated tissue was 47.33%.

**Figure 3:**
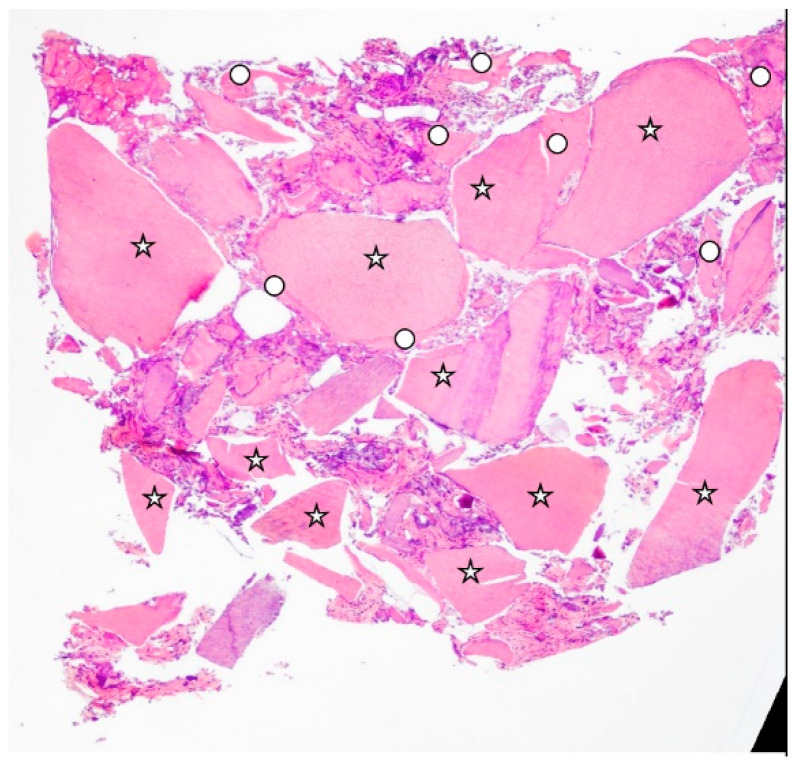
Histology extracted from Group 2 (dentin graft alone), With a circle for the bone and a star for the tooth graft. New bone was 4.12%, residual graft was 36.25%, and total calcified tissue was 43.49%.

**Figure 4:**
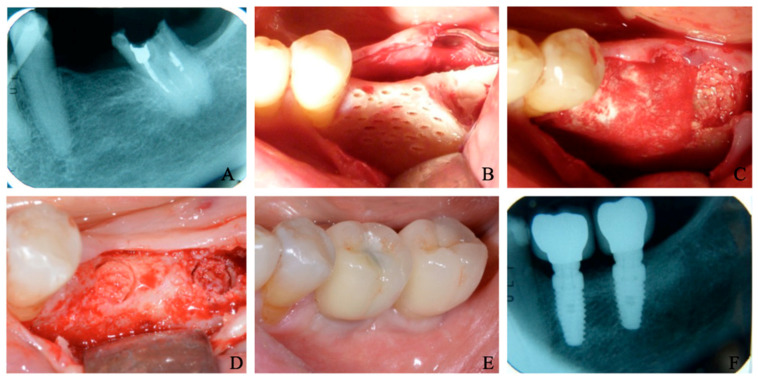
Clinical images of a case of alveolar ridge preservation with the two adjacent procedure types: (**A**) X-ray before the surgery; (**B**) Situation before alveolar ridge augmentation; (**C**) It is possible to note the membrane presence in site 3.6. Dentin graft alone is present in the position of tooth 3.7; (**D**) After 4 months, two biopsies were made on sites 3.6 and 3.7; (**E**) Definitive prosthesis; (**F**) X-ray after 6 months of load.

The results of the histomorphometric analysis (Table 1) of the graft biopsies showed that, in Group 1 (Figure 2), the mean BV% (bone volume) was 47.33 ± 2.48, the mean residual graft % was 5.65 ± 1.63, and the VB% (vital bone) was 41.67 ± 4.65. In Group 2 (Figure 3), the mean BV% was 37.34 ± 6.33, the mean residual graft % was 27.58 ± 15.42, and the VB% was 9.75 ± 11.81. In all cases, as a secondary outcome, it was observed that the regenerated bone allowed for the insertion of implants with a standard platform, implant stability quotient (ISQ) stability was enough to deliver a prosthesis for all cases (ISQ > 65, Table 2), and no early failures were recorded (Figure 4).

## 4. Discussion

**Figure 5:**
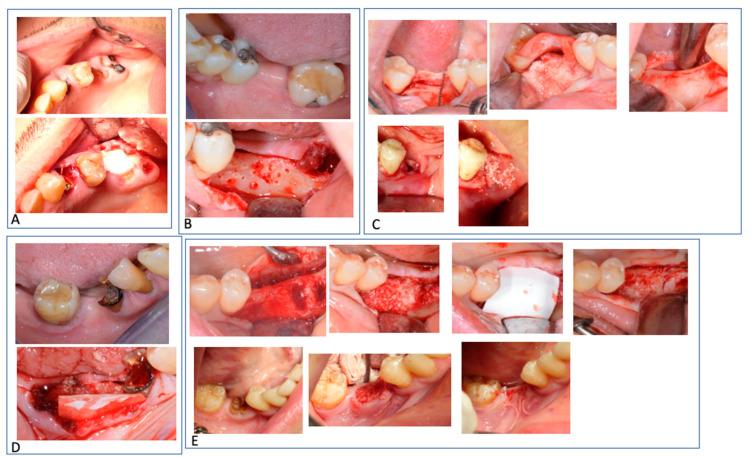
Images of all cases. Cases (**A**–**E**), the groups 1 and 2 are in different mouth sites.

Extracted teeth have long been considered waste materials. However, dentin is chemically and physically very similar to bone, with the only difference being that the latter is less mineralized [26]. The purpose of this case series was to define the differences between the use of an autogenous dentin particulate graft with and without collagen membrane after alveolar ridge augmentation procedures from a histomorphometric point of view (Figure 5).

Histomorphometric preliminary results demonstrated a significant amount of vital bone (Group 1: 38.42 ± 4.58 vs. Group 2: 9.75 ± 11.81) and bone volume (Group 1: 45.69 ± 2.31 vs. Group 2: 37.34 ± 6.33) in the case of membrane-associated ridge augmentation. On the contrary, the amount of residual graft is greater in cases of preservation of the socket associated with the exclusive use of vital graft (Group 1: 7.26 ± 2.28 vs. Group 2: 27.54 ± 15.42).

Some systematic reviews and meta-analyzes reported residual graft values of 12.4–21.11% in allograft cases. In cases where xenografts and alloplasts were used, the results were 37.14% and 37.23% at 7 months [27].

Therefore, it could be thought that the absence of a membrane creates a healing pattern of dentin grafts such as a xenograft. On the contrary, the use of membrane seems to enhance cell differentiation leading to extremely positive values at 4 months.

This result may lead us to speculate, which can only be clarified by further biochemical and molecular studies. In fact, the lack of new bone in the absence of a membrane could be determined by the greater speed of the fibroblastic line compared with the osteoblastic one, or by an inability to differentiate the mesenchymal cells into multinucleated giant cells that are responsible for the reabsorption of the graft.

Another very interesting aspect could be provided by the value of the ISQ in the regenerated sites 6 months after the loading of the implants. In fact, an optimal value for the delivery of the prosthesis was reached at all sites. This could lead to speculation that, in the sites without a membrane, the reabsorption of the graft in dentin may be slower or that the same phenomenon of preparation of the implant site can remodulate the resorption phenomenon of the residual graft, favoring the apposition of new bone.

Furthermore, a recent study based on 101 histological samples and 909 subsections showed that there is no histomorphometric correlation between the different types of defects and the recipient site. This further suggests that the results regarding the difference in regeneration quality between groups 1 and 2 are not related to the number of defect walls [28].

In 1967, Urist observed in mice that dentine grafts properly treated with 0.6 M HCl, sterilized, and washed in 70% alcohol, had a lower resorption rate than decalcified bone [29]. The first studies in this topic, however, explained how decalcified dentin could be highly biocompatible and resorbable [30]. Many methods have been proposed in the literature to decontaminate and make dentin more osteogenic. In fact, treatments with HCl, EDTA, hydrogen peroxide, ethanol, boiling in water, and gentamicin have been proposed [31]. The most interesting histological data is represented by the fact that the dentin, properly treated, can be used as a graft with low inflammatory properties. In fact, 5 days after implantation, the inflammatory reaction decreases, and the particulate is invaded by mesenchymal cells which are transformed into multinucleated giant cells that erode the graft. Subsequently, the matrix in which the graft is immersed is mineralized perhaps precisely due to the presence of the ionic residues of the reabsorption [32].

It has been shown that closed dentinal tubules generate chondrogenesis while open tubules stimulate osteogenesis, especially in granules between 420 and 850 µm. The osteoinductive properties of a dentin graft might depend on different acid treatment protocols, underlying the importance of this process [16].

Another important aspect is that the decalcification of dentin induces the release of the BMPs trapped in the hydroxyapatite and type I collagen matrix [33].

A new system for treating the dentin intended for grafting has recently been commercialized (TT Tooth Transformer srl. Milan, Italy).

The use of this system has shown some point of strength. In fact, this type of graft is totally autogenic, does not require an additional surgical site for harvesting bone graft, and the dentin structure and composition is very similar to that of bone. In addition, it has been shown that the product contains BMP-2, made available by the demineralization procedure. Therefore, this provides the material osteoinductive properties [22,23,24,25,26,27,28,29,30,31,32,33].

Certainly, future studies will be necessary to understand if it is possible to enhance gene expression and the osteoinduction of the dentin graft, also, through laser stimulation [34].

The physical–chemical and biochemical features of the dentin and enamel matrix obtained after processing teeth derivatives with the Tooth Transformer device have been described in a recent in vitro study [14,15,16,17,18,19,20,21,22,23,24,25]. A study [22] found that the biocompatibility of demineralized dentin is even higher than the more popular xenograft bovine bone, and that demineralization increases BMP-2 and type I collagen bioavailability as compared with non-demineralized tooth derivatives.

The design of this study involved the comparison of two groups to understand whether the presence of BMP-2 and type I collagen within the dentin graft could allow to simplify regenerative procedures and not use barrier membranes. The histomorphometry data showed us that the absence of a barrier membrane results in a quantity of new bone formed approximately 12 times less in the short term (4–6 months). An explanation of this phenomenon could be found by comparing the result with other studies on the subject. A review showed that all preliminary data regarding the Tooth Transformer method demonstrated histomorphometric data, like the results of this study, when a membrane is used [35,36,37].

One of the limitations of this experimental study is the short observation period. In the long term, various aspects can play a fundamental role in the preservation of clinical outcomes [38,39].

## 5. Conclusions

This study showed how the use of the membrane promotes better bone regeneration. Future studies with longer follow-up are needed to better evaluate whether the integration of implants in sites without a membrane will be stable over time. It will be necessary to investigate from a biological point of view why the autogenous tooth graft without a membrane had four times less quantity of bone and no clinical and functional differences were noted after implant loading at the early stages.

Autogenous dentin graft with a membrane has showed promising results with a high percentage of new vital bone around the residual graft material.

This suggested that the autogenous demineralized tooth graft obtained by the TT Transformer^®^ medical device can be considered a feasible, safe, and biocompatible alternative to other xenogeneic allogeneic biomaterials currently used in human alveolar socket augmentation procedures.

## Figures and Tables

**Table 1:** Histomorphometric parameters of Group 1 (dentin graft + membrane) and Group 2 (dentin graft alone).

	Group 1	Group 2
Bone Volume %	45.69 ± 2.31	37.34 ± 6.33
Residual Graft % (TT%)	7.26 ± 2.28	27.54 ± 15.42
Vital Bone %	38.42 ± 4.58	9.75 ± 11.81

**Table 2:** ISQ value at different times. No functional differences between the two groups (ISQ > 65 at 6 months from prosthesis load).

	Group 1	Group 2
ISQ Stability after Implants Healing	69.8 ± 3.87	62.1 ± 4.22
ISQ Stability after 6 months of Prosthesis Load	72.3 ± 1.19	65.9 ± 2.32
